# A plant pathogenic bacterium exploits the tricarboxylic acid cycle metabolic pathway of its insect vector

**DOI:** 10.1080/21505594.2017.1339008

**Published:** 2017-06-30

**Authors:** Nabil Killiny, Yasser Nehela, Faraj Hijaz, Christopher I. Vincent

**Affiliations:** a Citrus Research and Education Center, Department of Plant Pathology, IFAS, University of Florida, Lake Alfred, FL, USA; b Citrus Research and Education Center, Department of Horticultural Science, IFAS, University of Florida, Lake Alfred, FL, USA

**Keywords:** *Candidatus* Liberibacter asiaticus, *Diaphorina citri*, GC-MS, Huanglongbing, tricarboxylic acid cycle

## Abstract

Huanglongbing in citrus is caused by a phloem-limited, uncultivable, gram-negative α-proteobacterium, *Candidatus* Liberibacter asiaticus (*C*Las). *C*Las is transmitted by the phloem-sucking insect, *Diaphorina citri* (Hemiptera: Liviidae), in a persistent, circulative, and propagative manner. In this study, we investigated the metabolomic and respiration rates changes in *D. citri* upon infection with *C*Las using gas chromatography-mass spectrometry (GC-MS) and gas exchange analysis. The level of glycine, _L_-serine, _L_-threonine, and gamma-amino butyric acid were higher in *C*Las-infected *D. citri*, while _L_-proline, _L_-aspartic acid, and _L_-pyroglutamic acid were lower in *C*Las-infected *D. citri* compared with the control. Citric acid was increased in *C*Las-infected *D. citri*, whereas malic and succinic acids were reduced. Interestingly, most of the reduced metabolites such as malate, succinate, aspartate, and _L_-proline are required for the growth of *C*Las. The increase in citric acid, serine, and glycine indicated that *C*Las induced glycolysis and the tricarboxylic acid cycle (TCA) in its vector. In agreement with the GC-MS results, the gene expression results also indicated that glycolysis and TCA were induced in *C*Las-infected *D. citri* and this was accompanied with an increases in respiration rate. Phosphoric acid and most of the sugar alcohols were higher in *C*Las-infected *D. citri*, indicating a response to the biotic stress or cell damage. Only slight increases in the levels of few sugars were observed in *C*Las-infected *D. citri*, which indicated that sugars are tightly regulated by *D. citri*. Our results indicated that *C*Las induces nutrient and energetic stress in its host insect. This study may provide some insights into the mechanism of colonization of *C*Las in its vector.

## Introduction

Insect-transmitted plant pathogens have been intensely studied in recent years due to the large economic damage they cause. Insect vector-borne pathogens can be attributed to more than 700 plant diseases worldwide, which cause symptoms generally described as “scorch,” “blight,” “stunting,” and “yellows.”^1^ Specifically, insects of the order Hemiptera, such as psyllids, aphids, whiteflies, and leafhoppers, are largely responsible for transmitting many of these diseases.

In *Citrus* spp., Huanglongbing (HLB) also known as citrus greening, has rapidly dispersed across different geographical regions over the world and cost citrus growers billions of US dollars.^1^ HLB is caused by *Candidatus* Liberibacter spp, which are fastidious, phloem-limited, uncultivable, Gram-negative α-proteobacteria.^2,3^ Three *Ca.* Liberibacter species are associated with HLB; *Ca.* L. africanus (*C*Laf) in Africa, *Ca.* L. asiaticus (*C*Las) in Asia and Americas, and *Ca.* L. americanus (*C*Lam) in Brazil.^4^ Although *C*Las can be transmitted by graft inoculation, it is mainly transmitted by psyllids.^5^ The two psyllids associated with HLB-transmission are the African citrus psyllid, *Trioza erytrea* (Hemiptera: Triozidae) which transmits *C*Laf; and the Asian citrus psyllid, *Diaphorina citri* (Hemiptera: Liviidae), which transmits both *C*Las and *C*lam.^5^ *C*Las and the other Liberibacters are restricted to the phloem tissue of plants^6^ and therefore, the phloem sap must provide the required nutrients for the their multiplication.

Some hemipterans feed exclusively on plant phloem or xylem saps. The plant phloem sap is rich in sugars and organic acids, but usually low in essential amino acids.^7^ Usually, phloem-sucking insect consume large quantities of phloem sap and egest excess sugars in the form of honeydew.^7^ To complete the required balance of amino acids, most insects, including *D. citri*, harbor one or more bacterial endosymbionts which provide the missing nutrients.^7,8^

For insects to be able to host and transmit plant pathogens in persistent manner, the pathogen must be able to multiply within the insect hemocoel (propagative), circulate within the haemolymph and travel to the salivary glands, and finally be injected with saliva into a new plant host during feeding.^9^ In this type of transmission, the insect vector can transmit the pathogen for its whole life.^9^ *D. citri* transmits *C*Las in this manner.^10^ *D. citri* acquires *C*Las during feeding on the phloem sap of infected plants (acquisition), and the acquired pathogen is moved from the gut to haemolymph, then to the salivary glands and other tissues via haemolymph. Finally, *C*Las can be injected with the saliva into new tree phloem during subsequent feeding.^10^ *C*Las multiplies in both nymphs and adult psyllids^11^ indicating that the haemolymph of *D. citri* contains all the necessary nutrition needed for *C*Las propagation.^10^

Knowledge about pathogen-vector interactions is still limited, and is not without controversy. The specific interactions between plant bacterial pathogens and their hemipteran vectors remain poorly understood.^12,13^ These interactions may range from beneficial to harmful.^13^ Recently, Pelz-Stelinski and Killiny reported some mutually advantageous interactions in the case of the *C*Las-*D. citri* pathosystem. They found that *C*Las-infection increased the reproductive fitness of its vector.^13^ By contrast, in the same pathosystem, *C*Las-infection showed many harmful/negative effects on *D. citri* such as increasing the vector dispersal,^14^ susceptibility to insecticides,^15^ and decreasing its survival and life span.^13^ In addition, these interactions are difficult to study because many of the pathogenic bacteria have not been cultured yet.^16^

Metabolomics have become an important tool in the study of metabolites (small biological molecules) and has recently been applied to plants,^17^ insects,^18,19^ and bacteria.^20,21^ The non-targeted nature of metabolite profiling allows the analysis of the complete set of metabolites, commonly found in biologic fluids and tissues. The metabolomic profile of an organism can be affected by many factors including developmental stage, environmental factors, nutritional status, and biotic stresses.^22^ Previous studies showed that many metabolites were altered in the haemolymph of worker honeybees (*Apis mellifera* L.) upon their infection with the microsporidian *Nosema ceranae*.^22^ In addition, another metabolomic study revealed metabolic responses in silkworm after infection with the pathogenic fungus *Beauveria bassiana*.^23^ In the current study, we investigated the changes in *D. citri* metabolites upon infection with *C*Las using gas chromatography-mass spectrometry (GC-MS), and assessed the resulting impacts on insect respiration using gas exchange analysis. This study may provide insight into the mechanism of *C*Las colonization of its vector. In addition, the findings may give more information about how *D. citri* responds to and copes with *C*Las infection.

## Results

**Figure 1.**
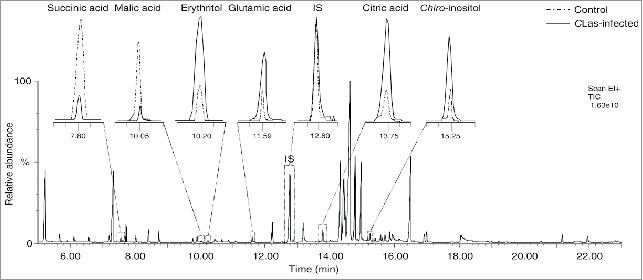
A representative gas chromatography-mass spectrometry (GC-MS) chromatogram of trimethylsilyl (TMS) derivatives detected in the extract of healthy and *Candidatus* Liberibacter asiaticus-infected *Diaphorina citri*, with a magnification of selected peaks.

Fifty-five metabolites were detected after TMS derivatization; 15 amino acids, 3 fatty acids, 7 organic acids, and 30 sugars and sugar derivatives (Table 1). A representative gas chromatography-mass spectrometry (GC-MS) chromatogram for the detected metabolites is shown in Fig. 1.

**Table 1.** Concentrations of different metabolic compounds (mean ± std) detected in Asian citrus psyllid, *D. citri* body after the infection with *C*Las using GC-MS.

Compound	Control (*n = 10*)	*C*Las-infected (*n = 40*)	*P*-value	Compound	Control (*n = 20*)	*C*Las-infected (*n = 20*)	*P*-value
**Amino acids** (ng insect^−1^)				**Sugars **(ng insect^−1^)			
_L_-Alanine	13.517 ± 6.259	24.587 ± 7.540	0.0048	Erthrulose	0.128 ± 0.039	0.201 ± 0.069	0.0038
_L_-Valine	0.186 ± 0.071	0.184 ± 0.063	0.9498	Lyxose	0.754 ± 0.100	1.249 ± 0.212	0.0000
Iso-Leucine	0.487 ± 0.137	0.778 ± 0.173	0.0015	Fructose	4.228 ± 0.337	6.079 ± 0.773	0.0000
_L_-Proline	0.708 ± 0.179	0.334 ± 0.093	0.0030	Mannose	0.028 ± 0.004	0.050 ± 0.009	0.0000
Glycine	3.860 ± 1.150	5.280 ± 1.625	0.0317	Glocose	9.129 ± 0.406	8.857 ± 1.079	0.3341
_L_-Serine	1.451 ± 0.170	2.401 ± 0.773	0.0000	β-Glucopyranose	0.019 ± 0.002	0.021 ± 0.003	0.2237
_L_-Threonine	1.333 ± 0.513	1.933 ± 0.446	0.0340	GlcNAc	0.129 ± 0.077	0.202 ± 0.117	0.0889
_L_-Aspartic acid	0.357 ± 0.059	0.207 ± 0.042	0.0009	Sucrose	0.124 ± 0.028	0.115 ± 0.022	0.4772
Glutamic acid	1.135 ± 0.329	1.958 ± 0.564	0.0004	Turanose	0.094 ± 0.003	0.090 ± 0.002	0.0553
_L_-Phenylalanine	0.163 ± 0.061	0.231 ± 0.167	0.1207	Trehalose	0.091 ± 0.003	0.096 ± 0.006	0.0093
_L_-Lysine	0.673 ± 0.382	0.563 ± 0.264	0.5284	Maltose	0.093 ± 0.003	0.096 ± 0.008	0.1263
Total	23.871 ± 6.850	38.456 ± 9.327	0.0015	Total	14.818 ± 0.669	17.057 ± 1.853	0.0001
**Non-proteinogenic amino acids** (ng insect^−1^)				**Sugars alcohol** (ng insect^−1^)			
_L_-Homoserine	0.047 ± 0.027	0.058 ± 0.029	0.4146	Erythritol	0.386 ± 0.017	2.060 ± 0.315	0.0000
Pyroglutamic acid	1.547 ± 0.288	0.399 ± 0.162	0.0001	Xylitol	0.305 ± 0.093	0.350 ± 0.065	0.3098
GABA	0.148 ± 0.022	0.402 ± 0.136	0.0000	Glucitol	0.254 ± 0.079	0.417 ± 0.199	0.0046
Putrescine	0.868 ± 0.261	0.864 ± 0.234	0.9766	Mannitol	0.524 ± 0.050	8.623 ± 0.711	0.0000
Total	2.610 ± 0.492	1.723 ± 0.273	0.0058	*Chiro*-Inositol	0.118 ± 0.017	0.352 ± 0.063	0.0000
**Organic acids** (ng insect^−1^)				*Scyllo*-inositol	0.241 ± 0.125	0.332 ± 0.107	0.1422
Pyruvic acid	0.006 ± 0.003	0.005 ± 0.002	0.2618	*Myo*-Inositol	3.380 ± 0.579	2.996 ± 0.920	0.2268
Lactic acid	0.011 ± 0.004	0.007 ± 0.003	0.0689	SA1	0.180 ± 0.034	0.237 ± 0.078	0.0139
Glycolic acid	0.003 ± 0.001	0.003 ± 0.000	0.3060	SA2	0.227 ± 0.074	0.300 ± 0.107	0.0793
Succinic acid	0.386 ± 0.080	0.185 ± 0.050	0.0011	Total	5.615 ± 0.654	15.668 ± 1.451	0.0000
Malic acid	0.910 ± 0.117	0.486 ± 0.132	0.0000	**Sugars acids** (ng insect^−1^)			
Threonic acid	0.446 ± 0.102	0.477 ± 0.106	0.5206	2-Ketoglutonic acid	0.103 ± 0.011	0.143 ± 0.043	0.0003
Citric acid	0.133 ± 0.035	2.633 ± 0.578	0.0000	2-Ketoglutaric acid	0.089 ± 0.010	0.085 ± 0.011	0.4484
Total	1.895 ± 0.291	3.796 ± 0.556	0.0000	Ribonic acid	0.244 ± 0.166	0.319 ± 0.211	0.3740
**Fatty acids** (ng insect^−1^)				Gluconic acid	0.162 ± 0.051	0.152 ± 0.041	0.6722
Palmitoleic acid	0.271 ± 0.083	0.197 ± 0.090	0.0919	Mannonic acid lactone	0.108 ± 0.023	0.138 ± 0.062	0.0663
Palmitic acid	0.083 ± 0.032	0.082 ± 0.035	0.9536	D-Glucuronic acid	0.055 ± 0.006	0.057 ± 0.013	0.6598
Oleic acid	0.251 ± 0.197	0.253 ± 0.200	0.9793	Total	0.760 ± 0.176	0.894 ± 0.245	0.1554
Total	0.605 ± 0.283	0.532 ± 0.275	0.5889				
**Phospho-compounds** (ng insect^−1^)							
Erythrose-4- phosphate	0.398 ± 0.111	0.378 ± 0.173	0.7318				
Glucose-6-Phosphate	0.0510 ± 0.0072	0.050 ± 0.010	0.8524				
Phytic acid	0.0514 ± 0.0065	0.046 ± 0.007	0.1327				
Phosphoric acid	17.913 ± 1.119	26.153 ± 1.827	0.0000				
Total	18.414 ± 1.217	26.628 ± 1.821	0.0000

### Amino acids

Eleven proteinogenic amino acids were detected in the derivatized psyllid samples and _L_-alanine was the most abundant amino acid (Table 1). Five of these amino acids were significantly affected by *C*Las. Glycine, _L_-serine, _L_-glutamic acid, and _L_-threonine were higher in *C*las-infected *D. citri*, whereas _L_-proline and _L_-aspartic acid were lower in *C*las-infected *D. citri* compared with the control (Table 1). The total concentration of proteinogenic amino acids was also increased by *C*Las infection (Table 1).

Four non-proteinogenic amino acids (NPAAs) were detected in the derivatized samples. *Gamma*-amino butyric acid (GABA) in *C*Las-infected *D. citri* was higher than the controls (Table 1). Pyroglutamic acid, was lower in *C*Las-infected *D. citri* compared with the control (Table 1). The total concentration of NPAA acids in *C*Las-infected *D. citri* was reduced by *C*Las infection (Table 1).

### Fatty and organic acids

Three fatty acids (palmitoleic acid, palmitic acid, and oleic acid) were detected in low amounts in the derivatized samples. None of these fatty acids was affected by *C*Las infection (Table 1). Seven organic acids were detected in the extract of *D. citri* (Table 1). Succinic and malic acid were lower in *C*Las-infected *D. citri*, whereas citric acid was higher (Table 1). As a result of the tremendous increase in citric acid, the total level of organic acids was increased in *C*Las-infected psyllids (Table 1).

### Sugars, sugar alcohols, sugar acids, and phosphorous compounds

Eleven sugars were detected in the TMS-derivatized psyllid extracts (Table 1). Glucose was the most abundant sugar followed by fructose (Table 1). Erythrulose, lyxose, fructose, trehalose, and mannose were slightly higher than the control (Table 1). The disaccharide (turanose) in *C*Las-infected psyllids was lower than the controls (Table 1). The remaining sugars were not affected (Table 1). The total concentration of sugars was also slightly affected upon *C*Las infection (Table 1).

Nine sugar alcohols were detected in the TMS-derivatized samples and *myo*-inositol was the most abundant sugar alcohol in uninfected psyllids (Table 1). Erythritol, *chiro*-inositol and mannitol in *C*Las-infected psyllids were higher than the controls (Table 1). As a result of the increase in most of sugar alcohols, the total concentration of sugar alcohols was also increased in *C*Las-infected *D. citri* (Table 1).

None of the sugar acids was affected by *C*Las infection, except 2-ketoglutonic acid which was slightly increased (Table 1). Phosphoric acid was significantly higher in *C*Las-infected compared with the control (Table 1). As a result of the high increase in phosphoric acid, the total concentration of the phospho-compounds in *C*Las-infected psyllids was higher than the controls (Table 1).

### Principal component analysis (PCA)

**Figure 2.**
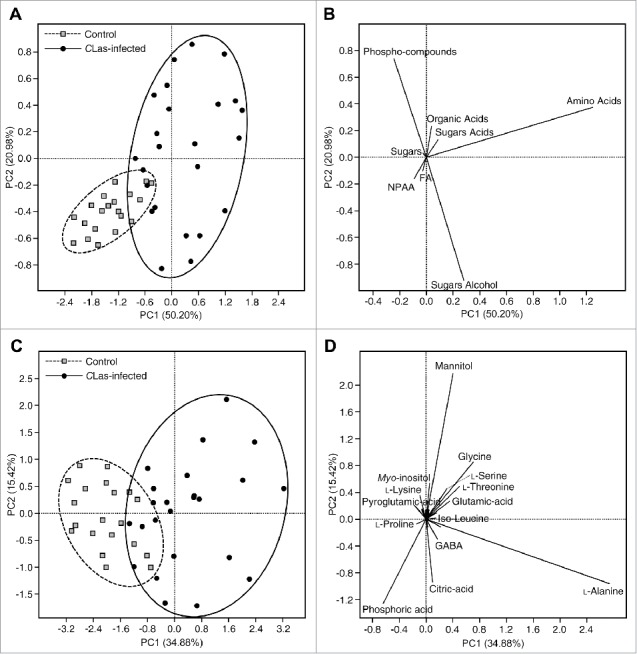
Principal components analysis (A) and its associated loading plot (B) showing the distribution of uninfected and *Candidatus* Liberibacter asiaticus-infected *Diaphorina citri* using the concentrations of the main metabolites groups. Principal components analysis (C) and its associated loading plot (D) showing the distribution of uninfected and *Candidatus* Liberibacter asiaticus- infected *Diaphorina citri* using the concentrations of individual metabolites groups (n = 20). In panel (D), Some of the detected compounds names have been deleted from the loading plot for better presentation.

The PCA of *C*Las-infected and uninfected psyllids are shown in Fig. 2A-D. The PCA of *C*Las-infected and uninfected psyllids generated using the total concentration of the main groups is shown in Fig. 2A and B. Principal component 1 and 2 explained about 71.18% of the variation. As shown in the score plot **(**Fig. 2A), the uninfected psyllids were almost separated from the *C*Las-infected psyllids. The loading plot showed that the *C*Las-infected psyllids were higher in most of the metabolite groups, whereas the uninfected psyllids were higher in NPAA (Fig. 2B).

The PCA of *C*Las-infected and uninfected psyllids generated using the concentration of all detected metabolites (Table 1) is shown in Fig. 2C and D. Principal component 1 and 2 explained about 50.30 % of the total variation. The uninfected psyllids were partially separated from the *C*Las-infected psyllids (Fig. 2C). These results indicated that the metabolite profile of *C*Las-infected psyllids was different from that of uninfected psyllids. The loading plot (Fig. 2D) visualized the distribution of the detected metabolites among the *C*Las-infected and uninfected psyllids. As shown in Fig. 2D, *C*Las-infected psyllids were higher in metabolites that appeared in the right of plot such as citric acid, erythritol, *scyllo*-inositol, mannitol, GABA, and phosphoric acid. On the other hand, uninfected psyllids were higher in metabolites that appeared in the left of the plot such as _L_-proline, pyroglutamic acid, succinic acid, lactic acid, and malic acid (Fig. 2D).

### Effect of *C*Las on the genes expression of *D. citri*

**Figure 3.**
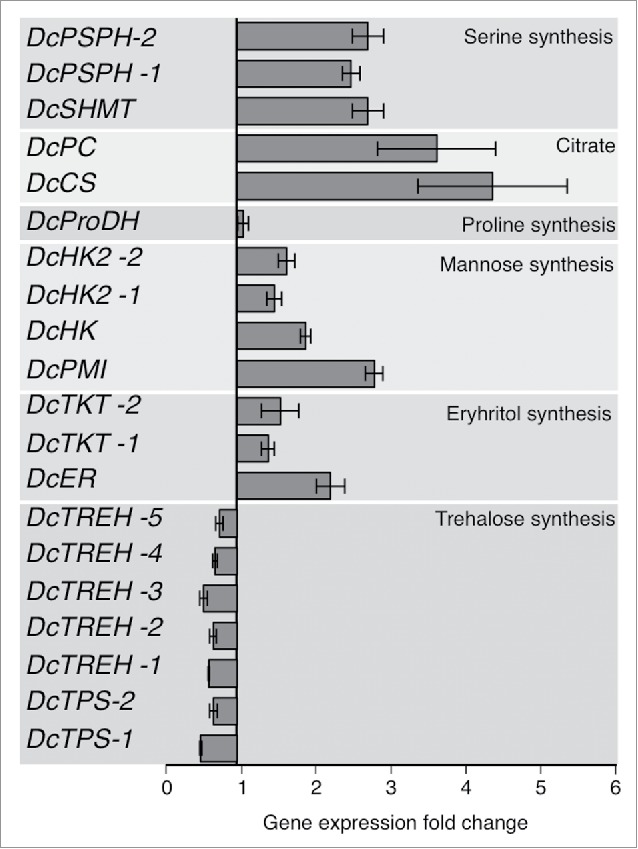
Differential gene expression patterns of expressed genes involved in TCA cycle of *C*Las-infected and uninfected *D. citri*. The complete list of expressed genes is available Table 2.

The gene expression of citrate synthase (*DcCS*) and pyruvate carboxylase (*DcPC*), which are implicated in the synthesis of citric acid, were highly upregulated (5.1 ± 1.2 and 4.2 ± 0.95 fold change, respectively) in *C*Las-infected psyllids (Fig. 3). In addition, the expression of hexokinases (*DcHK, DcHK-1*, and *DcHK-2*), which catalyze the phosphorylation of hexoses, was upregulated in *C*Las-infected psyllids (Fig. 3). The gene expression of enzymes implicated in serine and glycine (*DcPSPH* and *DcSHMT*) biosynthesis were also upregulated (2–3-fold) in *C*Las-infected psyllids (Fig. 3). The level of the gene expression of mannose-6-phosphate isomerase (*DcPMI*) was increased in *C*Las-infected psyllids (Fig. 3). The gene expression of the enzymes implicated in erythrose biosynthesis (*DcER* and *DcTKT*) were slightly upregulated in *C*Las-infected psyllids. On the other hand, genes involved in trehalose metabolism such as α, α trehalose phosphate synthase (*DcTPS*) and trehalase (*DcTREH*), were expressed at a lower level (0.42 ± 0.02 and 0.47 ± 0.06, respectively) in infected-*D. citri* (Fig. 3).

### Respiration

**Figure 4.**
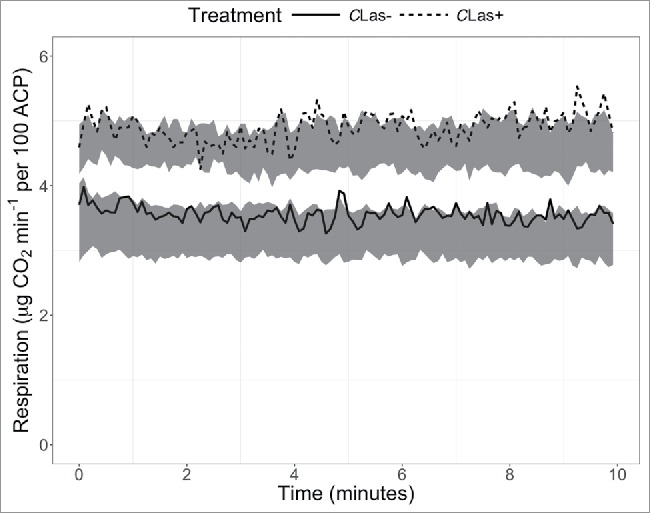
Respiration of *C*Las-infected and uninfected *D. citri* recorded over 10 min. Gray bands represent 95% confidence intervals (n = 6), and lines represent single representative samples.

Respiration rates of *C*Las-infected *D. citri* were higher than those of uninfected *D. citri* (*F* = 25.9, *P* = 0.007; Fig. 4), with the mean respiration rate of *C*Las-infected *D. citri* being 1.34 µg CO_2_ min^−1^ higher than that of uninfected *D. citri* (42% increase). The coefficient of variation was not affected.

## Discussion

*C*Las-infection has tremendous effects on its host plants, resulting in mottled leaves, twig dieback and slow growth, lopsided and bitter or sour fruits, and finally results in tree decline and death.^24^ In addition, the *C*Las pathogen significantly affects the primary and secondary metabolites in its host plants.^25-27^ In this work, we focused on the effects of *C*Las on the *D. citri* metabolites.

The levels of many amino acids were altered in *D. citri* as a result of *C*Las infection. Our results showed that glycine, _L_-serine, and _L_-threonine were significantly higher in *C*Las-infected *D. citri* compared with the controls. In agreement with the GC-MS results, the gene expression of enzymes involved in glycine and _L_-serine were also upregulated in *C*Las-infected psyllids. Because glycine and serine can be converted to 3-phosphoglycerate and fed into the glycolysis pathway, the increase in these 2 amino acid indicated an induction in the glycolysis pathway in *C*Las-infected *D. citri*. The increase in glycolysis rate is also supported by the gene expression results which showed an increase in the gene expression of enzymes implicated in glycolysis such as hexokinase and pyruvate carboxylase. On the other hand, our current results also showed that _L_-aspartic acid was reduced in *C*Las-infected *D. citri*. The reduction in _L_-aspartic acid may also result from its oxidation to supplement the TCA cycle intermediates (fumarate and oxaloacetate) which would be under higher demand under the energetic stress caused by *C*Las infection. In addition, the reduction in _L_-aspartic acid could result from its consumption by *C*Las which cannot synthesize it and relies on exogenous _L_-aspartate.^28^

Our results also showed that the levels of proline in *C*Las-infected *D. citri* was lower than that of the controls. The reduction in proline may result from the reduction in its biosynthesis or the increase in its consumption or both. Proline biosynthesis in the haemolymph could be reduced as a result of high demand for Acetyl-CoA for the TCA cycle. Proline could also be catabolized to produce energy in *C*Las-infected psyllids and this suggestion is supported by the increase in respiration rate. Previous studies showed that proline was the major source of fuel for flight muscles in some insects.^29^ The decrease in proline in *C*Las-infected psyllids could result, at least in part, from its consumption by *C*Las and this suggestion is supported by the genome analysis which showed that *C*Las is incapable of producing proline.^28^

Our results also showed that GABA was higher in *C*Las-infected psyllids compared with the control. GABA is widely distributed in plant and animals^30^ and it was also detected in the citrus phloem sap and haemolymph of *D. citri.*^31,32^ The level of GABA in plants increases under various environmental stresses such as acidosis, mechanical damage, anoxia, heat and cold shock, drought, salt, and fungal, viral, and insect attack.^30,33^ The high level of GABA in *C*Las-infected *D. citri* could result from its feeding on infected host plants. However, it also possible that glutamic acid decarboxylase (GAD) enzymes is activated in *C*Las-infected *D. citri* to increase the production of GABA which can also be converted to succinate and fed in the TCA cycle. On the other hand, pyroglutamic acid was reduced in *C*Las-infested *D. citri*. The decrease in pyroglutamic acid could result from the activation of 5-oxoprolinase, which convert pyroglutamte to glutamate, as a result of high demand for glutamate to produce GABA. The increase in _L_-glutamic acid supports the previous assumption.

Our results showed that citric acid was higher in *C*Las-infected psyllids compared with the controls. The increase in citric acid level in in *C*Las-infected psyllids is in agreement with the gene expression results, which showed an increase in the expression of hexokinases, pyruvate decarboxylase, and citrate synthase, a regulatory enzyme in TCA cycle. In agreement with our results, a previous proteomic study showed that many enzymes involved in metabolism and cellular energy storage and utilization were induced at a higher rate in *C*Las-infected *D. citri*.^34^ These enzymes included several enzymes involved the citric acid cycle and such as succinate dehydrogenase, 2-oxoglutarate dehydrogenase (a regulatory enzyme in TCA cycle), and L-2-hydroxyglutarate dehydrogenase.^34^ Both Acetyl-CoA dehydrogenase and enoyl-CoA hydrolase proteins enzymes were also induced (2-fold and 12-fold, respectively) in *C*Las-infected *D. citri*.^34^ These 2 enzymes are involved in fatty acid β-oxidation and the production of Acetyl-CoA which feeds into the TCA cycle.^34^ Glycerol kinase enzyme which is required for triglyceride breakdown was also upregulated in *C*Las-infected *D. citri*.^34^ In addition, 2 glycolysis enzymes (aldose 1-epimerase and phosphoglycerate mutase) were induced in *C*Las-infected *D. citri*.^34^ Succinate semialdehyde dehydrogenase which converts succinate semialdehyde, produced from GABA, to succinate was also induced in *C*Las-infected *D. citri*.^34^ The metabolomics and the proteomic results together suggested that glycolysis and TCA cycle are enhanced in *C*Las-infected *D. citri*. In addition, a previous transcriptome study also showed that *C*Las infection alters the expression of many genes involved in nutrient reservoir activity in *D. citri*.^35^ Gene expression of *C*Las-infected *D. citri* suggested that *C*Las alters its host environment to import needed nutrients.^35^

Although the current gene expression and the previous proteomic analysis confirmed that most of the enzymes involved in the citric acid cycle were induced in *C*Las-infected psyllids,^34^ the level of malate and succinate in *C*Las-infected psyllids were lower than the control. This result indicated that *C*Las may acquire succinate and malate from *D. citri*. Since isocitrate lyase and malate synthase are absent in *C*Las genome, it is believed that *C*Las lack the glyoxylate bypass.^28^ Therefore, *C*Las depends on exogenous fumarate, malate, succinate, and aspartate as carbon substrates.^28^ These intermediates could also be exhausted by *D. citri* which is under the energetic stress due to *C*Las infection.

The activation of TCA cycle in *C*Las-infected psyllids indicated that infected psyllids are under nutrient and energy stress. Previous studies showed that *C*Las encodes an ATP/ADP translocase in its genome, indicating that *C*Las can import exogenous ATP.^36,37^ Recently, we studied the effect of *C*Las on the nucleotides profile of *D. citri* and found that ATP and many other nucleotides were increased upon *C*Las infection.^38^ The accumulation of ATP in *C*Las-infected *D. citri* psyllids increased the adenylated energy charge (AEC) and decreased the AMP/ATP and ADP/ATP ratios. In agreement with the increase in AEC, the survival and life span of *C*Las-infected psyllids were also lower than those of healthy psyllids.^38^ In addition, electropenetrography showed that *C*Las increased the feeding activity of *D. citri* indicating that *C*Las-induced energetic stress in its vector.

The increases in glycolysis and the TCA-cycle of *C*Las-infected *D. citri* likely account for increased respiration rates. Although there are potential biochemical pathways to decouple aging from respiratory rates,^39^ increased respiratory rates in other insect species are generally associated with reduced lifespan,,^40,41^ and increased respiratory rates are considered to be tradeoffs with other fitness characteristics.^42^ This is consistent with existing literature regarding the impact of *C*Las on *D. citri* that has found that *C*Las infection increases feeding activity, dispersal, and reproductive fitness, but reduced nymphal development rate and lifespan.^13,14^ Many of these changes may be influenced by increased respiration resulting from *C*Las manipulation of *D. citri* energetics metabolic pathways.

Although our results showed that the glycolysis and TCA pathways were induced in *D. citri* upon *C*Las infection, only a slight increase in trehalose, mannose, xylose, and fructose was observed in *C*Las-infected *D. citri*. This result indicated that the mechanism regulating sugar levels in the *D. citri* was not highly affected by *C*Las infection. Because carbohydrates are the main source of energy in insects, their levels must be maintained at a high level to support flight muscle and other activities.^22^ In general, sugars content is very high in the phloem sap, therefore phloem sap-feeding insects should be able to overcome the sugar barrier which can cause high osmotic pressure.^7^ A slight decrease in fructose and increase in glucose was observed in honeybee worker haemolymph infected with *Nosema ceranae*, whereas the level of trehalose was not affected.^22^ The previous results together with our result indicated that sugars are under tight regulation in insects. In fact, previous studies showed that trehalose synthesis is inhibited when trehalose concentration reaches a certain level and UDP-glucose is directed for glycogen synthesis.^43^ The increase in glycogenin protein in *C*Las-infected *D. citri* suggested that extra trehalose in *C*Las-infected *D. citri* is being converted to glycogen.^34^

Sugar alcohols (inositols) were previously reported in the citrus phloem sap^44^ and the haemolymph.^32^ Several roles for polyols in insects have been proposed including the regulation of osmotic pressure, protection against cold and heat stress, and meeting the nutritional requirements of insect symbionts like yeast.^45^ The increase in sugar alcohols in *C*Las-infected *D. citri* could be a response to the biotic stress caused by *C*Las infection. Phosphoric acid was also higher in *C*Las-infected *D. citri*. Phosphoric acid was also increased in honeybee worker (*Apis mellifera* L.) haemolymph upon infection with *Nosema ceranae*.^22^ Our current results together with the previous result indicated that the increase in phosphoric acid could be a general indicator of infection or cell damage.

In conclusion, our results showed that *C*Las alters the metabolomic profile of its vector and enhanced the glycolysis and TCA cycle. In agreement with the increase in the glycolysis and TCA cycle, the respiration rate of *C*Las-infected psyllids was induced. Our current results indicated that *C*Las-infected psyllids are under nutrients and energetic stress and this could explain their increased feeding and flight activity and reduced lifespan. Changes observed in this study could reveal insights into *C*Las pathogenicity.

## Materials and methods

### Asian citrus psyllid culture

Colonies of Asian citrus psyllid, *Diaphorina citri*, were reared on uninfected (control) or *C*Las-infected Valencia sweet orange (*Citrus sinensis* ‘Valencia’). Both colonies as well as the citrus plants were constantly monitored with PCR^5^ to confirm infection with *C*Las. Adult of healthy (control) and *C*Las-infected psyllids were sampled without age or gender discrimination from healthy and *C*Las-infected colonies. The collected *C*Las-infected psyllids were kept on uninfected plants for 2 weeks before metabolite extraction to avoid the possible effects of poor plant quality on *D. citri* metabolism. Colonies were kept in growth rooms which were set at 27–28°C, 60–65% relative humidity (RH), and a photoperiod of 14L:10D h.

### Metabolite extraction

To improve the detection limits of the *D. citri* metabolites, we relied on whole-organism sample pooling. Groups of 50 *D. citri* adults were collected from *C*Las-infected (58 ± 6.0%) and healthy colonies (control). Ten replicates of 50 (500 total) insects from healthy *D. citri* adults (never been exposed to a *C*Las-infected plant) and 40 replicates of 50 (2000 total) insects from *C*las-infected *D. citri* adults (reared on *C*Las-infected plants) were collected. The collected *D. citri* adults were placed in – 20°C for 2 h (cold anesthetized) to facilitate handling. Insects were transferred into 1.5 mL microcentrifuge tubes. To extract a broad range of metabolites, a 100 μL aliquot of the extraction solvent (8:1:11; methanol: chloroform: water) was added to each tube of insects. Insects were macerated by homogenizing with a motorized pestle in the extraction solution for 5 min. Tubes of macerated insects were placed on ice and a glass bead was added to each tube. Samples were then placed on an Ocelot platform mixer (Model 260300F, Boekel Scientific, Feasterville, PA) and rocked overnight at 4°C. After overnight extraction, the tubes of insect homogenate were vortexed briefly and centrifuged at 5°C for 5 min at 10, 000 rpm in a refrigerated centrifuge (Model 5430 R, Eppendorf, Hauppauge, NY) to remove solid debris. The supernatant was collected from each tube and placed into new 1.5 mL tubes.

### Derivatization, GC-MS conditions and peak analysis

Ten μL aliquot of each *D. citri* supernatant was placed into a silanized GC vial with 200 μL fused-insert (MSCert4000–30LVW, National Scientific, Rockwood, TN) along with 10 μL of internal standard (1000 ppm ribitol in water, Sigma Aldrich, St. Louis, MO). This solution was dried under a nitrogen stream before adding the derivatization reagents. Methoxyamine hydrochloride solution (MOX) in pyridine (2%), and N-methyl-(N-trimethylsilyl) trifluoracetamide (MSTFA), were purchased from ThermoFisher Scientific (Waltham, MA). To the dried samples, 30 μL MOX was added and incubated at 60°C for 1 hour. Finally, 80 μL MSTFA was added and incubated an additional 1 h at 60°C. The derivatized samples were injected splitlessly into the GC injector (1 μL) for analysis using the same GC-MS column and conditions described by Killiny (2016).^46^ Total ion chromatograms were analyzed using Turbomass software and peaks of interest were identified based on the relative retention time and ion spectrum comparison of authentic standards to sample spectra and by using Wiley 9th ed. and NIST 2011 mass spectral libraries. Calibration curves for authentic standards were constructed from 4 classes of compounds, amino acids, organic acids, sugars and sugar alcohols derivatized in the same manner as the samples. For compounds in which a reference standard was not available, quantification was based on the standard curve of the reference compound most similar in chemical class and relative concentration. Compounds present in sufficient quantity were converted from peak area to μg g^−1^ FW insect concentration. Concentrations (ng/insect) reported are the mean of 10 biologic replicates.

### Respiration

Uninfected (control) and *C*Las-infected (58 ± 6.0%) *D. citri* were collected from rearing cages in a contained growth chamber after at least 2-hours from the beginning of artificial daytime. Approximately 100 individuals at a time were placed in a respiration chamber. The chamber was allowed to warm to ambient temperature, approximately 24˚C, for 25 min before beginning measurements. The insect respiration was measured using an infrared gas analyzer (LI-6400; Licor Biosciences, Inc.; Lincoln, NE, USA) at a flow rate of 500 µmols min^−1^. Because insect respiration measurements depend on the dynamics of movement, measurements were recorded every 5 sec for 16.6 min for each sample.

Sampling was performed in replicate sets of *C*Las-infected and healthy psyllids. Two replicates each were tested each day for 3 d. After respiration measurements, the number of insects were counted, and insects were killed and fresh weight measured, followed by drying for 24 hours at 70˚C and recording of dry weights. Respiration was weighted by the number of insects, and analyzed as µg CO_2_ per 100 *D. citri*.

### Gene expression analysis using quantitative real time PCR (RT-PCR)

The total RNA was extracted from 10 insects per replicate (3 replicates for each treatment), using TriZol® reagent (Ambion®, Life Technologies, NY, USA). Both quantity and quality of isolated RNA were determined using NanoDrop 2000 spectrophotometrer (Thermo Scientific, USA). For synthesising cDNA, SuperScript first-strand synthesis system (Invitrogen) with random hexamer primers was used as described by the manufacturer's instructions. Further, the qPCR was preformed using SYBER Green PCR master mix (Applied Biosystems), on an ABI 7500 Fast-Time PCR System (Applied Biosystems). Samples were analyzed in triplicate for each biologic replicate for each treatment. Primers for 29 selected genes, involved in the affected cycles of *D. citri* (increase or decrease in its compounds), were used to measure the gene expression (Table 2). The 2^−ΔΔ^*^C^*T method was used to determine the relative expression of the consensus sequence among PCR products according to Livak and Schmittgen (2001).^47^ Gene expression data was normalized using α-Tubulin or actin as endogenous reference genes as described by Tiwari et al. (2011) and Killiny et al.(2014).^48,49^

**Table 2.** Primers used in gene expression analysis of selected genes of *D. citri* by real time RT-PCR^a^.

Gene	NCBI Reference Sequence		Primer (Forward and Reverse)	TM (˚C)	Product size (bp)
*DcTPS* -1	XM_017448460.1	F	AGTCATTATCCAGGGCAACG	59.96	198
		R	CAGAAGGTGCCATTACAGCA	59.86	
*DcTPS* -2	XM_008470475.1	F	CCTACATCCGGATTGACCAG	60.33	192
		R	CGTACAGTTGGCGAATTCCT	60.13	
*DcTREH* -1	XM_008470849.1	F	GGTAATGGAGGCGAGTACGA	60.10	190
		R	ACAGGAACAGGATGCCTCAC	60.12	
*DcTREH* -2	XM_008470850.2	F	TCACCTCCAACCCGAGTATC	59.93	191
		R	CAGTTCTCGAAACCGTCCTC	59.84	
*DcTREH* -3	XM_008476679.2	F	CTGAAGTGTGTGCTCCGAAA	60.02	197
		R	GAGTCGGGAAACAGTGAAGC	59.85	
*DcTREH* -4	XM_008476680.2	F	TGCAGTTCAGATGGCTTCAC	59.99	198
		R	GGCGACCAGTCTTCAAACTC	59.85	
*DcTREH* -5	XM_008476684.2	F	GCAGTGGGACTACCCTAACG	59.62	195
		R	CCGTGTCCTCCTGGTACTGT	60.03	
*DcER* -1^b^	XM_008489926.2	F	GAGAGGTGAAACAGGCGGTA	60.25	200
		R	TGCAGGTTTCACGAGATCAG	59.98	
*DcTKT* -1	XM_008486923.2	F	CCACGCACAGAGACATCATT	59.71	201
		R	CGGTGGTCGGTACAGATTTT	59.85	
*DcTKT* -2	XM_008471702.2	F	CCAGATCCGTATGGGAGCTA	60.05	201
		R	CCTGGGATTGTACGGAACAT	59.67	
*DcMPI*	XM_008474470.2	F	CCAGAATTGGCCATAGCACT	60.10	199
		R	GAGCTTGGGGAGCACTCATA	60.36	
*DcHK*	XM_008477526.2	F	AAGGATAGACCGGGATGACA	59.36	199
		R	CTGGAAAGCGCCTAGTTTCA	60.51	
*DcHK2* -1	XM_008480214.1	F	GCGAGCTGGTTCGTGTAGTA	59.10	200
		R	ATCCACCGCTATGGTGATGT	60.23	
*DcHK2* -2	XM_017444207.1	F	CTAGCCGGCCTAGGATTACC	60.08	202
		R	TCATCAGGTCATGGAAGTGC	59.64	
*DcProDH*	XM_017446986.1	F	GAAGGTGACCAAGGATGCTC	59.66	199
		R	TCGGACCAGGGTGTTTAGTC	59.97	
*DcCS*	XM_008470046.1	F	TGCCCGATGACTCCTTATTC	60.04	190
		R	CCAAGAGCTCTGGACACTCC	59.99	
*DcPC*	XM_008478621.1	F	GAAGCTTTGGCTTCATTTGG	59.82	201
		R	GTCGCGGACACTTACACTCA	59.90	
*DcSHMT*	XM_008473310.2	F	TCGAACCTCATGGGAGAATC	60.01	197
		R	TCCTTTGTGAGTGGTGGTCA	60.13	
*DcPSPH*-1	XM_008483178.2	F	GGCCAAAGTCATCGAGAGTC	59.81	202
		R	GACCACGTTGCCTCCATAAC	60.38	
*DcPSPH*-2	XM_008472446.2	F	TGAACCCGGAGCAGATATTC	60.04	209
		R	ACTCAGGCTCGGCTTCATAA	59.98	
*α-Tubulin*^c^	XM_008482608.2	F	CTTTCCAACACCACCGCTAT	59.99	196
		R	CTCCTTCTCCAGCCTCCTCT	60.09	
*Actin*^c^	XM_008470468.2	F	TATCCCAGCCCTGAGCTAGA	59.93	204
		R	CACCATGACACCCTGATGAC	59.80	

a The listed genes were selected depending on the affected cycles (increase or decrease in its compounds). Furthermore, these genes were assembled based on recent available data in national center for biotechnology information website (NCBI, http://www.ncbi.nlm.nih.gov/gene/).

b *DcER* gene has been matched as *Diaphorina citri* aldose reductase-like (*DcAR*) using the protein-protein BLAST, based on recent available data in national center for biotechnology information website (NCBI, http://www.ncbi.nlm.nih.gov/gene/).

c Genes have been used as a reference genes for data normalization according to Tiwari *et al.*, 2011^48^ and Killiny *et al.*, 2014^49^

*Abbreviations*:

***DcTPS***-1: *Diaphorina citri*-α, α Trehalose phosphate synthase;

***DcTPS***-2: *Diaphorina citri*-Trehalose phosphate synthase-like;

***DcTREH***: *Diaphorina citri*-Trehalase;

***DcER***: *Diaphorina citri*-Erythrose reductase;

***DcTKT***: *Diaphorina citri*-Transketolase-like;

***DcMPI***: *Diaphorina citri*-Mannose-6-phosphate isomerase;

***DcHK***: *Diaphorina citri*-Hexokinase-like;

***DcHK2***: *Diaphorina citri*-Hexokinase-2-like;

***DcProDH***: *Diaphorina citri*-proline dehydrogenase 1;

***DcCS***: *Diaphorina citri*-probable citrate synthase 2;

***DcPC***: *Diaphorina citri*-pyruvate carboxylase, mitochondrial-like;

***DcSHMT***: *Diaphorina citri*-serine hydroxymethyltransferase, cytosolic;

***DcPSPH***: *Diaphorina citri*-phosphoserine phosphatase-like

### Statistical analysis

Data was manually aligned using retention time and mass values. The concentrations of each metabolites in *C*Las-infected *D. citri* were compared with the control using *t*-test. Principal component analysis (PCA) was performed on individual metabolite concentrations to discriminate healthy and *C*Las-infected psyllid using JMP version 9.0 (SAS Institute Inc.,). The PCA was also conducted using the total concentrations of the main groups of metabolites shown in Table 1. Analysis of variance was performed on the mean and coefficient of variation of each insect respiration sample (mean of 200 measurements each) for the purpose of testing overall differences in respiration rates (mean) and of testing the relative variation in rates (coefficient of variation). Analysis used a linear model in R statistics package (R Foundation, Vienna, Austria) with Treatment (*C*Las-infected or healthy *D. citri*) as a fixed effect and replicate as a random effect.
